# The Effects of Passive Smoking on the Six-Minute Walk Test in Obese Pediatric Cases

**DOI:** 10.4274/jcrpe.1524

**Published:** 2014-12-05

**Authors:** Nazan Kaymaz, Şule Yıldırım, Mustafa Tekin, Hakan Aylanç, Fatih Battal, Naci Topaloğlu, Fatih Binnetoğlu, Ayla Akbal

**Affiliations:** 1 Çanakkale Onsekiz Mart University Faculty of Medicine, Department of Pediatrics, Çanakkale, Turkey; 2 Çanakkale Onsekiz Mart University Faculty of Medicine, Department of Physical Medicine and Rehabilitation, Çanakkale, Turkey

**Keywords:** children, obesity, passive smoking, walking test

## Abstract

**Objective:** The aim of this study was to evaluate whether exposure to second-hand smoke affected the six-minute walk test (6MWT) of obese non-asthmatic pediatric cases.

**Methods:** Obese pediatric patients (body mass index >95th p) with no existing co-morbidities were included in the study. Smoke exposure was assessed with a self-reported questionnaire completed by the parents. The subjects were divided into two groups: Group 1 consisting of obese children exposed to passive smoking and Group 2 of obese children not exposed to passive smoking. In addition to 6MWT, spirometric flow and volume, including forced expiratory volume in 1 s and peak expiratory flow rate, were also measured in all subjects. The results of the 6MWT were assessed to determine any association with passive smoking.

**Results:** The study included 75 obese pediatric cases (40 male, 35 female) with a mean age of 9.06±0.97 years. The 6MWT results in Group 1 was 501.88±62.12 meters and in Group 2 559.63±72.93 meters. The difference was statistically significant (p=0.001).

**Conclusions:** Passive smoking may negatively affect the respiratory and cardiovascular capacity in obese children, who are already at risk of lower cardiopulmonary function. The evaluation of 6MWT in these pediatric patients may be useful for monitoring and families should be warned about potential problems due to smoking.

## INTRODUCTION

The leading preventable health risks today are smoking and obesity. The incidence of several cardiovascular risk factors including high blood pressure, dyslipidemia and metabolic syndrome, is linked to childhood exposure to tobacco. Obesity also exerts its negative effects on arterial structure via increased intima-media thickness and function through decreased elasticity and endothelial function (1). In the past thirty years, the rate of obesity has increased fourfold in children aged 6-11 years (2). Endothelial dysfunction and increased cardiovascular morbidity and mortality are linked to habits and states leading to insulin resistance, such as smoking and obesity (3). Overweight or obese children are at higher risk of being affected by existence of smoking in the family (4). Being a passive smoker is known to be nearly as harmful as being an active smoker. Exposure to environmental tobacco smoke affects children most, as children breathe more frequently than do adults and thus inhale more environmental tobacco smoke (5). The implementation of laws against smoking in the workplace and public areas has left the home as the dominant unregulated source of exposure to smoking. This has significant effects on children who cannot remove themselves from the environment filled with tobacco smoke (6).

Decreased fitness and decreased aerobic capacity have been linked to increased weight in adults (7). Impaired cardiopulmonary status in obese pediatric patients can also limit the functional capacity and have negative effects on ability to partake in exercise. The six-minutes walk test (6MWT) is a simple, practical, standardized, reliable and valid measure of effect of submaximal exercise in healthy children (8,9,10). The test reflects the state of cardiopulmonary fitness in addition to state of physical activity in daily life and is considered to be the most relevant walk test (8). Disability in obese subjects has been reported in previous studies using the 6MWT (11).

The aim of this study was to determine whether exposure to passive smoking affected the 6MWT results in non-asthmatic obese pediatric cases.

## METHODS

This case-control study was conducted in the time period between December 2013 and May 2014 at Çanakkale Onsekiz Mart University Hospital. In this study, we enrolled a total of 75 children (M/F=40/35) with a mean age of 9.06±0.97 (min:7-max:10) years with a diagnosis of exogenous obesity and no co-morbidities including hypertension, impaired cardiac function, glucose intolerance, hyperlipidemia, hypothyroidism. Exclusion criteria were also neuromuscular disorders and ataxia, mental retardation, cerebral palsy, systemic disease affecting the respiratory tract directly or indirectly, chronic or acute disease affecting the respiratory tract directly or indirectly, use of inhaled corticosteroids, bronchodilators or other medication affecting the respiratory tract, history of recent or chronic respiratory disease, history of severe respiratory disease, i.e., congenital lung disease, hospitalization for pneumonia or thoracic surgery. Three children with hypertension and 2 children with glucose intolerance were subsequently excluded. To ensure compliance, only children over the age of 7 were included in the study. The etiology of obesity in the patients had been previously investigated and follow-up was being conducted by the pediatric obesity clinic. Our hospital is a tertiary center, which explains its higher prevalence of obesity as compared to the general population. This is one reason why we chose to perform the present study in our hospital.

Exogenous obesity was defined as obesity not linked with any endocrine, metabolic or genetic causes. The parents of all children were asked to answer a questionnaire comprising the child’s demographic characteristics and his/her history of exposure to passive smoking.

The participants were then divided into two groups. Group 1 consisted of obese children exposed to smoking and Group 2 of obese children who were not exposed to smoking. None of the participants were reported to have followed a regular exercise program. The study was approved by the Çanakkale Onsekiz Mart University Research Ethics Committee and all parents provided a written informed consent.

**Anthropometric Measurements and Body Mass Index (BMI)**

A stadiometer, calibrated daily, was used to measure body weight and height for all patients (Seca 703, accurate to 100 g, Seca GmBH&Co Kg; Hamburg, Germany). The same investigator (BG) measured body weight and height in all children. Weighing was done with the children wearing only underwear, no external clothing. Height was measured by standard measurement in a standing position. BMI was calculated using the formula weight (kg)/height (m)2. Taking the standard reference percentiles for Turkish children, patients with a BMI value above the 95th percentile or a BMI standard deviation score (SDS) above +2.0 SD were evaluated as obese (12).

**Exposure to Passive Smoking**

Parental smoking habits were assessed according to the information provided by the parents. Consumption of five or more cigarettes per day over a period of five years or more by mother and/or father was assessed as “exposure to passive smoking” for the purposes of this study (13). None of the children smoked and primary school children were included in the study specifically to prevent inclusion of active smokers. Tager (14) considered the use of a questionnaire insufficient to assess passive smoking and urinary cotinine levels were measured for support. However urinary cotinine levels have been suggested to be inadequate for accurate assessment (14,15). Our study assessed passive smoking with a questionnaire.

**6MWT, Spirometric Flow and Volume Measurement Procedures**

6MWT and lung function were assessed on the same day, in the same place and at the same environmental temperature (18 ˚C) in all subjects. The subjects had spirometric flow, forced expiratory volume in 1 second (FEV1) and peak expiratory flow rate (PEFR) measured with a portable mini-Wright digital peak flowmeter (HS Clement Clarke International, UK) for two consecutive days. The spirometer did not require repeated calibration. Before and after 6MWT, after resting for five minutes in a sitting position, all lung functions were measured again. Three expiratory flow maneuvers were performed for FEV1 and PEFR. The study included the highest measurements recorded. Patients were asked to complete PEF as quickly as possible and continue for ≥2 s. If the patient coughed, the blow was too short or had a slow start, they were asked to repeat the procedure. The FEV1 and PEFR results in our cases were compared with pulmonary functions in healthy Turkish children from a study performed by Arslan et al (16) in 1993. In this study, pulmonary function test parameters showed no significant relationship with age, height and weight. Our pulmonary function test results were also compared with data from another previous study (17). If FEV and PEF were below the value predicted for age, height and gender (n=2), the children were excluded from the study. While encouraging the patient to achieve peak lung function values, the same experienced investigator provided all instructions and the patient was accompanied by at least one parent during the instruction. 6MWT was completed in accordance with the guidelines of the American Thoracic Society (18). The children were told to walk on flat ground around two flag poles 30 m apart in a standardized protocol. They were told to walk at a quick steady pace without running for 6 minutes. They were informed when 5 minutes had passed. No other verbal commands or feedback was given. The exact distance was measured by the trained study investigator who oversaw the test. Two walking trials were held before the test. The actual test began 5 minutes after the last trial. The patients were told that if required they could stop. In such a case, the stopwatch would continue and the test would be completed in 6 minutes or intervention would occur to save the test results. No patient stopped during the test.

**Data Analysis**

The data were analyzed using the SPSS version 20.0 software (SPSS Inc.). Descriptive statistics were performed to summarize sample characteristics. Normal distribution of data was assessed using the Kolmogorov-Smirnov test. Continuous variables are shown as mean±SD or median (min-max), where applicable. The mean differences between groups were compared with the student’s t-test, while the Mann-Whitney U test was applied for comparisons of median values. Categorical data were analyzed by Pearson’s chi-square or Fisher’s exact test, where appropriate. A p-value of less than 0.05 was considered statistically significant. 

## RESULTS

Most of the children in the series were born at term with a birth weight between 2500 and 3500 g (min:2000-max:4900) (Table 1). Three patients were born small for gestational age and 4 patients were born large for gestational age.

Mean six-minute walking distance in meters (m) (6MWD) was 501.88±62.12 meters in Group 1 and 559.63±72.93 meters in Group 2. The difference was statistically significant (p=0.001).

With the exception of 2 patients in Group 1 and 2 patients in Group 2, all subjects were breastfed. Duration of breastfeeding in the two groups is given in Table 1. When all patients in the study were evaluated, 6MWD had a positive correlation with the duration of breastfeeding (r=0.345, p=0.02).

Female patients in Group 1 had shorter 6MWD results than the girls who were not exposed to passive smoking and the difference was statistically significant (Table 2).

In Group 1, 26 mothers smoked (mean duration 11.4 years) and 31 fathers smoked (mean duration 15.9 years). For smokers, including both mothers and fathers, minimum and maximum smoking duration was 5 and 20 years, respectively. Passive smoking status of the parents and its association with 6MWD are given in Table 3.

6MWD values of children with both parents who smoked were found to be significantly shorter than those with only mothers who smoked (p=0.046) (Mann-Whitney U test).

## DISCUSSION

Our study demonstrated that obese pediatric patients who were exposed to passive smoking had shorter 6MWD than obese children who were not. Passive smoking is a well-known risk factor for reduced respiratory capacity (19). Our study population was investigated with this in mind and patients with normal respiratory capacity were included in the study. Passive smoking causes antioxidant depletion increasing systemic inflammation and sensitivity to other oxidant stresses (20), which is already increased in obesity (21). Coexistence of obesity and passive smoking may increase oxidant stress. On the other hand, in a recent study by Pavic et al (22), the children of smoking parents were found to have higher BMI than children of non-smoking parents. In the same study, 6MWT was also performed on children of smoking and nonsmoking parents and impairment of physical condition, especially in children with both parents smoking, was shown. Our study population consisted of children who were already obese and the BMI values were higher in children exposed to tobacco smoke than in those who were not, but the difference was not statistically significant. Our study also showed that 6MWD was shortest for children whose parents both smoked. In the passive smoking group, 6MWD of only mothers who smoked was significantly longer than those where both parents smoked.

Adverse respiratory effects are more strongly linked to paternal smoking in infants when the mother does not smoke, according to Cook and Strachan (23). Blackburn et al (24) reported that the major contributor to total tobacco consumption in a household is the father. These results may be due to infants spending more time with the mother than the father. 6MWD was found to be mainly dependant on age (25). In our study also, the younger children (those aged 7 years) were more affected compared to other ages and this group was also the group most affected by exposure to smoke. This may be due to the increased time spent by younger children in the company of parents. After the age of seven, the time spent in school limits the time in the home, leading to less exposure to smoke. However, there was no correlation between duration of exposure and 6MWD in our correlation analysis. We believe further studies with larger populations and older age children will further clarify the difference due to duration of exposure.

Current guidelines state that the 6MWD is influenced by sex (18). 6MWD values showed no differences between girls and boys in the present study. However, our study results demonstrated that girls who were exposed to tobacco smoke had shorter 6MWD than girls who were not.

6MWD had a positive correlation with duration of breastfeeding in this study, indicating that it is both prophylactic and preventive. Lower rates of obesity are linked to breastfeeding, which has a protective affect against obesity in later life (26). Yilmaz et al (27) found that breastfeeding promoted the growth of infants who were passively exposed to tobacco smoke and it is advised that breastfeeding should be promoted to protect infants against the health hazards of passive smoking. It primarily protects against obesity and secondarily prevents cardiopulmonary complications in obese patients.

To the best of our knowledge, this is the first study evaluating the effect of passive smoking on 6MWT in obese pediatric cases. Passive smoking in obese children, already a risk group, may result in greater decline in cardiovascular and pulmonary function. Evaluating 6MWT in these patients may be useful for monitor and families should be warned about potential problems due to smoking. While smoking is a modifiable risk factor, to reduce the obesity-related disease burden exposure to passive smoking should be avoided to improve the health of these patients by educating parents, especially through public health programs.

**Acknowledgements**

To the best of our knowledge, no conflict of interest, financial or other, exists.

## Figures and Tables

**Table 1:**
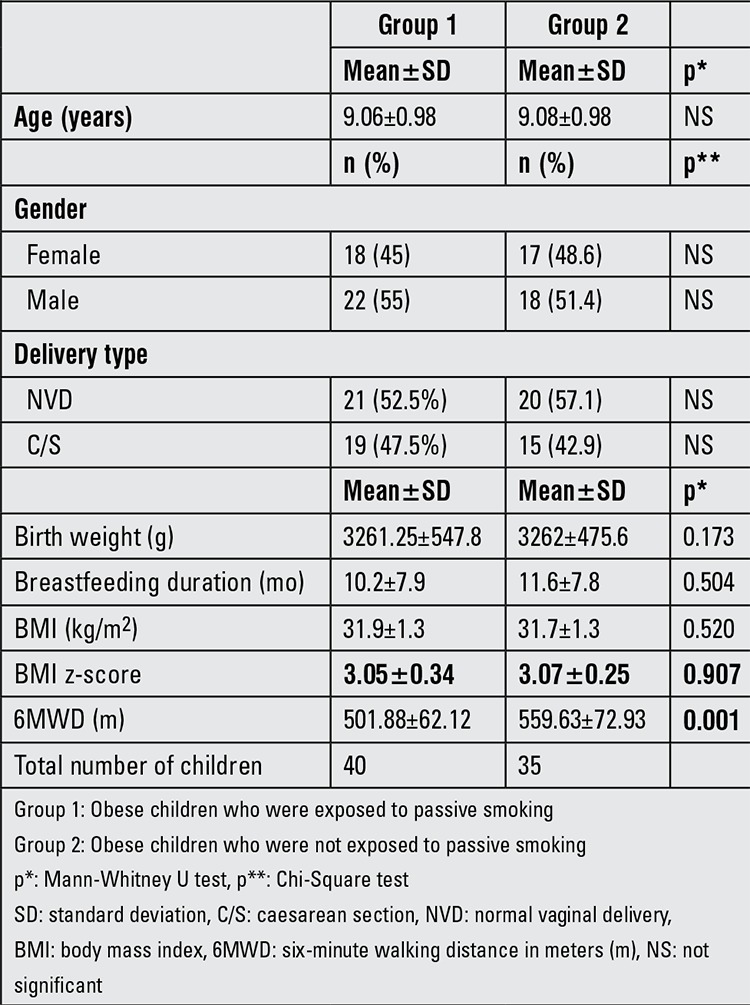
Patient characteristics of the groups

**Table 2:**
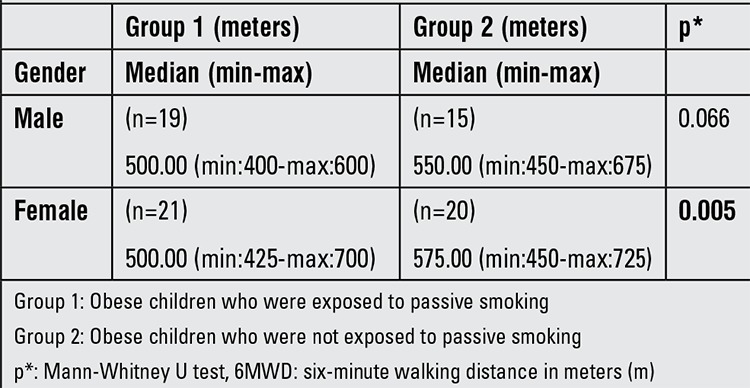
6MWD according to gender in the two groups

**Table 3:**
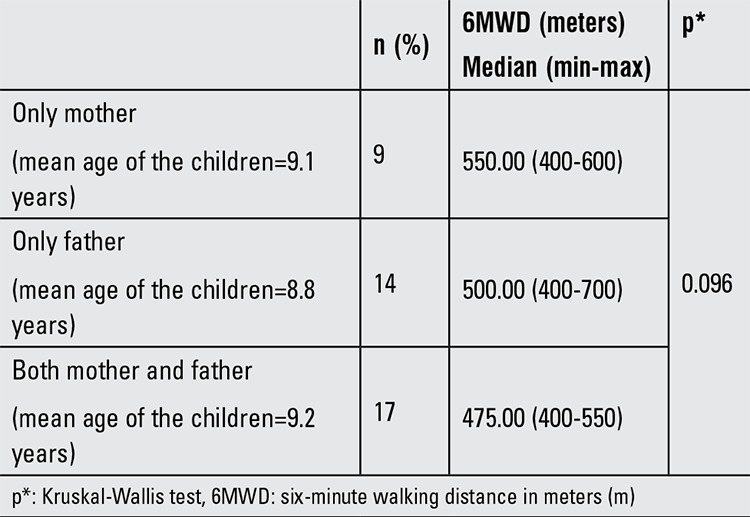
Passive smoking status of the parents and median 6MWD results in Group 1
